# Variation between Ethiopian and North American barley varieties ( *Hordeum vulgare* ) in response to Russian wheat aphid ( *Diuraphis noxia* ) populations

**DOI:** 10.1093/jis/14.1.40

**Published:** 2014-01-01

**Authors:** Alemu Araya, Tesfay Belay, Temam Hussein

**Affiliations:** 1 Debre Tabor University, Bahirdar, Ethiopia; 2 Tigray Agricultural Research Institute, P.O. Box 492, Mekelle, Tigray, Ethiopia; 3 Haramaya University, Haramaya, Ethiopa

## Abstract

The Russian wheat aphid, *Diuraphis noxia* (Mordvilko) (Hemiptera: Aphididae), causes severe damage to barley, *Hordeum vulgare* L. (Poales: Poaceae), in the highlands of Ethiopia. Little information is available on the control of this pest in Ethiopia. An experiment aimed at evaluating the resistance of barley varieties from the USA to *D. noxia* populations and determining biotypic variation between Ethiopian and North American *D. noxia* populations was conducted. The *D. noxia* -resistant barley varieties Burton and RWA-1758 from the USA, the resistant barley line 3296-15 from Ethiopia, and a local Ethiopian susceptible variety were included in a randomized design in a greenhouse under natural light conditions. There were highly significant differences ( *P* < 0.001) in the mean *D. noxia* population, leaf chlorosis, leaf rolling, plant stunting, number of tillers per plant, and the percentage of infested tillers per plant between the resistant and susceptible varieties. The aphid population per tiller was lower on the resistant barley plants than on the susceptible plants. Severe plant damage was observed on the local barley variety, while the least damage was observed on Burton, followed by RWA-1758. Burton and RWA-1758 were therefore highly resistant and moderately resistant, respectively, to the northern Ethiopian *D. noxia* populations, indicating similarities in biotypes between the United States and northern Ethiopian *D. noxia* populations. The damage to variety 3296-15 was greater than to Burton and RWA-1758. Leaf chlorosis scores and leaf rolling scores for variety 3296-15 upon treatment with the north Ethiopian *D. noxia* population indicate likely biotypic variation between *D. noxia* populations of northern and central Ethiopia.

## Introduction

Barley, *Hordeum vulgare* L. (Poales: Poaceae), is one of the earliest and most widely cultivated cereal crops grown in the highlands of Ethiopia ( Pohlman 1959 ). In the *meher* (main) season, it is the fifth most common cereal crop after maize, sorghum, tef, and wheat in terms of area coverage and total production (CSA 2010). In the *belg* (short) season, barley is the second most common cereal crop, after maize, in terms of area coverage and total production (CSA 1996). It is the major cereal crop in the highlands of north Ethiopia during all seasons. The crop is grown in diverse ecological conditions with an altitude range of 1,800 to 3,400 m a.s.l. ( Lakew et al. 1993 ).

Barley yields are very low in Ethiopia, but many people believe that barley has a high yield potential ( Lakew et al. 1993 ). Barley yields in Ethiopia were 1.33 and 0.96 t ha ^-1^ in the *meher* and *belg* seasons, respectively. This is very low compared to the potential maximum yield of 13.3 t ha ^-1^ reported by other sources ( FAO 1994 ). The major reason for the low yield is that the crop is produced under numerous constraints, including insect pests. A total of 38 insect pest species that attack barley were listed by Haile and Ali (1985) . The Russian wheat aphid, *Diuraphis noxia* (Mordvilko) (Hemiptera: Aphididae), is the major insect that reduces barley yields, and it has a worldwide distribution including the Middle East, USA, South Africa, and Ethiopia ( Haile and Ali 1985 ; Girma et al. 1993 ; Robinson 1994 ).

In Ethiopia, *D. noxia* has been a serious barley pest for about two decades ( Mulatu and Gebremedhin 1994 ). Currently, *D. noxia* is a major pest in all barley growing regions of Ethiopia, especially those at altitudes above 2,500 m a.s.l. where barley is the major food crop and is cultivated throughout the year. Miller and Haile (1988) reported yield losses of 41–79% in barley due to this pest and up to 86% in wheat in Ethiopia. In South Africa, losses in wheat yields of between 21 and 92% were reported ( Hewitt 1988 ). Calhoun et al. (1991) also reported yield losses of up to 59% in barley in Mexico.

The use of resistant cultivars is the ideal management option for *D. noxia* ( Robinson 1992 ). *D. noxia* -resistant barley lines were also identified in Ethiopia. Biotypic variation can threaten the durability of host plant resistance to insects ( Saxena and Barrion 1987 ), and biotypes develop as a result of selection from the parental population in response to exposure to resistant cultivars or other pressures. Fifty percent of recognized insect biotypes on crops belong to the family Aphididae ( Saxena and Barrion 1987 ). Puterka et al. (1992) first identified biotypes of *D. noxia* in worldwide collections. Smith et al. (2004) observed biotypic differences between North Ethiopian and American *D. noxia* populations, and since then, seven biotypes have been identified in the USA alone ( Randolph et al. 2009 ).

It is therefore necessary to monitor the biotypic status of *D. noxia* in Ethiopia as a component of a comprehensive integrated pest management approach. Thus, this study was initiated with the following objectives: 1) to evaluate the resistance of barley varieties to northern Ethiopian *D. noxia* populations; 2) to determine the biotypic differences between the Ethiopian and North American *D. noxia* populations through an evaluation of resistant barley varieties.

## Materials and Methods

### Description of the study area

The greenhouse experiment was carried out at the Mekelle Agricultural Research Center, which is located at 13°5'0'' N, 39°6'0'' E. The elevation is 1,970 m a.s.l. The area is situated in the semiarid agro-ecological zone of the region, which is characterized by low and erratic rainfall (Legesse 1999). Annual rainfall ranges from 445 to 550 mm, while the annual temperature range is between 12.2 and 26.5°C. The soil type is mainly clay loam with a pH of 7.47. The major crops grown in the area are wheat, barley, teff, and chickpea.

### Experimental design and treatments

The study was conducted in a greenhouse under natural light conditions. Two *D. noxia* -resistant barley varieties from the USA (Burton and RWA-1758) ( Bregitzer et al. 2005 ; Bregitzer et al. 2008 ), a *D. noxia* -resistant barley line from Holetta Research Centre (3296-15) ( Mulatu et al. 1993 ), and a susceptible local variety ( *saesa* ) from northern Ethiopia were included in the experiment. Three seeds from each variety were placed at a depth of 2.5 cm in a plastic pot filled with a medium composed of silt, sand, and manure in a 2:1:1 ratio. After emergence, the seedlings were thinned to one plant per pot. The temperature in the greenhouse ranged from 25–29°C, and the relative humidity was 60–70%. The plants were infested with approximately 20 *D. noxia* adults at Zadok’s leaf stage three ( Zadok et al. 1974 ). The *D. noxia* were placed on each plant with a soft brush. Infested plants were immediately covered with cages, plastic cylinders with ventilation holes on two sides. The holes were covered with fine mesh that allowed easy entry of air but prevented the movement of the aphids from one plant to another. The tops of the cages were also covered with fine mesh. The height and diameter of the plastic cages used were 25 and 9 cm, respectively. The area cut out of the plastic cage and covered with the muslin cloth was 4.5 cm x 5 cm (22.5 cm ^2^ ). The *D. noxia* were obtained from nearby barley fields that were planted one month before the start of the experiment. Care was also taken to carefully select *D. noxia* to avoid parasitism. The experimental design was randomized and included four replicates. Separate replicates of infested plants were maintained for scoring stunting. The plants were examined for aphid damage 14 days after being infested.

The data collected included leaf chlorosis, leaf rolling, plant stunting, *D. noxia* population count, the number of tillers per plant, and the percentage of infested tillers per plant. Chlorosis was recorded visually using a 0–9 scale ( Webster et al. 1987 ), where 0 = immune, 1 = plants appear healthy, 2 = isolated chlorotic spots are prominent, etc., and 9 = plants are dead or damaged beyond recovery. Leaf rolling was recorded on a scale of 1–3 ( Webster et al. 1987 ) where 1 = unfolded, 2 = one or more leaves are conduplicately folded, and 3 = one or more leaves are convolutely folded. Plant stunting was recorded on a scale of 1–5 ( Burd et al. 1993 ) where 1 = non-stunted and 5 = highly stunted.

The data were analyzed using the GenStat 12 ^th^ edition statistical software ( GenStat 2009 ). The percentage of infested tillers were square roottransformed, and the analysis of variance procedure was employed. Least significant difference tests were also used to separate the means whenever found significant.

## Results and Discussion

The results from the analysis of variance for leaf chlorosis, leaf rolling, plant stunting, *D. noxia* population count, the number of tillers, and the percentage of infested tillers per plant of the tested genotypes are presented in Table 1 . Significant differences were evident between the barley varieties for all of the variables.

**Table 1.**
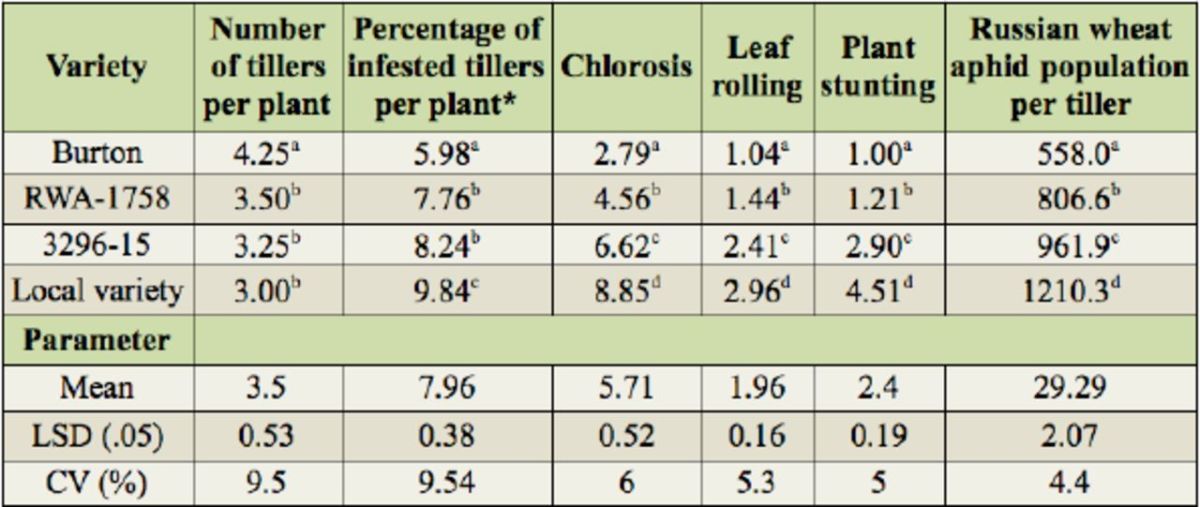
The number of tillers per plant, the percentage of infested tillers per plant, chlorosis score, leaf rolling score, plant stunting score, and Russian wheat aphid population per tiller for the tested barley varieties grown in a greenhouse.

Means in the same columns followed by the same letter are not significantly different ( *p* > 0.001). Chlorosis was recorded visually using a 0– 9 scale ( Webster et al. 1987 ), where 0 = immune, 1 = plants appear healthy, 2 = isolated chlorotic spots are prominent… and 9 = plants are dead or damaged beyond recovery. Leaf rolling was recorded on a scale of 1–3 ( Webster et al. 1987 ) where 1 = unfolded, 2 = one or more leaves are conduplicately folded, and 3 = one or more leaves are convolutely folded. Plant stunting was recorded on a scale of 1–5 ( Burd et al. 1993 ) where 1 = non-stunted and 5 = highly stunted. LSD, least significant difference.

*The mean percentage of infested tiller values are square roottransformed values. The original mean percentage infested tillers per plant numbers were 35.3, 59.7, 67.4, and 96.3 for Burton, RWA-1758, 3296-15, and the local variety, respectively.

There were significant differences ( *P* < 0.001) in chlorosis between the resistant and the susceptible barley varieties. The local susceptible barley variety had large chlorotic streaks and a higher chlorotic score than the resistant varieties. The lowest chlorosis score was observed for Burton, and the next lowest was for RWA-1758 ( Table 1 ). Burton was highly resistant, while RWA-1758 had intermediate resistance to the northern Ethiopian *D. noxia* populations, likely indicating similarities in biotypes. According to Bregitzer et al. (2005 , 2008), Burton and RWA-1758 are resistant to damage caused by two and five *D. noxia* biotypes, respectively, recorded in the USA. The major component of resistance was tolerance.

The chlorosis score for barley line 3296-15 was higher than that of Burton and RWA-1758, but it was lower than that of the susceptible local barley variety. According to Mulatu et al. (2011) , barley line 3296-15 had a low leaf chlorosis score of 4.33 upon treatment with the central Ethiopian *D. noxia* populations. In this study, however, an increased leaf chlorosis score was recorded with the northern Ethiopian *D. noxia* population, indicating likely biotypic variation between the northern and central Ethiopian *D. noxia* populations. Belay and Stauffer (2010) also recoded a cholorsis score of 5.75 for variety 3286-15 treated with the northern Ethiopian *D. noxia* population. *D. noxia* infestation symptoms are well documented ( Saheed et al. 2007 ; Tolmay et al. 2007 ) and often result in reduced effective area, chlorophyll content, and photosynthetic ability of the leaves ( Walters et al. 1980 ; Fouche et al. 1984 ).

Minimal chlorosis is often associated with resistance, as several researchers (Heng-Moss et al. 2003: Wang et al. 2004 and Botha et al. 2005) did not report significant changes in leaf color (chlorosis) or a reduction in photosynthetic activity for resistant cereal hosts. Botha et al. (2005) further reported on the maintenance of the chloroplast machinery as a determining factor in enabling resistant varieties to overcome stress during *D. noxia* feeding. Similarly, *D. noxia* feeding on susceptible cereal hosts caused a significant decrease in the total chlorophyll content ( Burd and Burton 1992 ), altered chlorophyll fluorescence induction kinetics, and reduced photochemical efficiency of photosystem II ( Burd and Elliott 1996 ).

The mean leaf rolling of the tested barley varieties upon treatment with *D. noxia* is presented in Table 1 . Leaf rolling was high on the susceptible barley variety, and this response was significantly different from those of the other barley varieties. Burton had flat leaves and the lowest leaf rolling score, which was significantly different from that of the other barley varieties, although the scores of RWA-1758 and Burton were similar. Additionally, barley line 3296-15 had a leaf rolling score that was significantly different from the scores of the other varieties.

Barley line 3296-15 had a leaf rolling score of 3.17 on the 0–9 scale in an experiment conducted at Holetta Research Center using central Ethiopian *D. noxia* (Mulatu et al. 2008). The increased leaf rolling score of 2.41 (moderately susceptible) on the 1–3 scale of Webster et al. (1987) in our study could indicate biotypic variation between the *D. noxia* populations of central and northern Ethiopia. *D. noxia* feeds on host plants in dense colonies within tightly curled leaves preventing the normal unrolling of newly emerging leaves ( Hewitt et al. 1984 ; Riedell 1989 ). Leaf rolling is observed as an almost immediate phenotypic response in the susceptible varieties after *D. noxia* feeding (Botha and Lapitan, unpublished).

The mean plant stunting scores for the tested barley varieties are presented in Table 1 . There was a significant difference ( *p* < 0.01) in stunting between the susceptible and resistant barley varieties. Burton and RWA-1758 exhibited almost the same minimal plant stunting response, and in agreement with this result, these varieties are more resistant to the northern Ethiopian *D. noxia* population. Variety 3296-15 exhibited less plant stunting compared with the local susceptible barley variety. According to Burd et al. (1993) , plant stunting best predicted the damage by *D. noxia* infestations in oats, wheat, and triticale; susceptible germplasm were more stunted than resistant varieties.

Differences in *D. noxia* population density ( *p* < 0.001) were observed between the resistant and the susceptible barley varieties ( Table 1 ). The highest population of *D. noxia* per tiller was recorded on the local susceptible barley variety, and the lowest populations were recorded on Burton and RWA-1758, respectively ( Table 1 ). Therefore, the number of aphids per tiller was drastically decreased on the resistant varieties compared to the susceptible varieties. The population density on variety 3296-15 was higher compared to Burton and RWA-1758, but much lower than that of the susceptible local barley variety. Similar results were reported by Michel et al. (1994), who also found significantly more Russian wheat aphids per plant on susceptible barley varieties.

The percentages of infested tillers per plant for the tested barley varieties are also presented in Table 1 , and highly significant differences ( *p* < 0.001) were detected among the varieties. Burton had the lowest percentage of infested tillers. There was no difference in the percentage of infested tillers between RWA-1758 and barley line 3296-15. The local variety had the highest percentage of infested tillers. Mornhinweg (1994) also reported a lower percentage of damaged tillers by *D. noxia* on the resistant compared to the susceptible varieties. Similarly, *D. noxia* feeding typically reduced the leaf number in susceptible cereals ( Burd et al. 1993 ).

## Conclusion

The number of the aphids per tiller was drastically decreased on the resistant barley varieties compared to the susceptible varieties. There were also significant differences in mean leaf chlorosis, leaf rolling, and plant stunting between the resistant and the susceptible varieties. The local variety sustained severe damage, while the least damage was observed on Burton and RWA-1758. Therefore, Burton and RWA-1758 are resistant to the northern Ethiopian (Tigray) *D. noxia* populations. This result indicates similarities in biotypes between the USA and the northern Ethiopian *D. noxia* populations. While seven biotypes have been identified in the USA so far ( Randolph et al. 2009 ), this study did not identify the biotypes in Ethiopia.

The plant damage (chlorosis, rolling, and stunting) for line 3296-15 was higher compared to Burton and RWA-1758, but lower than that of the susceptible local barley variety. Variety 3296-15 was resistant to central Ethiopian *D. noxia* . In this study, however, increased leaf chlorosis and leaf rolling were recorded upon treatment with the northern Ethiopian *D. noxia* population, indicating likely biotypic variation between the *D. noxia* populations of northern and central Ethiopia. There is therefore a need for further study on the biotypes in Ethiopia using known *D. noxia* biotypes for comparisons.
